# Inhaled biologics for respiratory diseases: clinical potential and emerging technologies

**DOI:** 10.1007/s13346-025-01909-6

**Published:** 2025-07-14

**Authors:** Nur Adania Shaibie, Nur Dini Fatini Mohammad Faizal, Fhataheya Buang, Teerapol Srichana, Mohd Cairul Iqbal Mohd Amin

**Affiliations:** 1 Centre for Drug Delivery Technology and Vaccine, Faculty of Pharmacy, Universiti Kebangsaan Malaysia, Jalan Raja Muda Abdul Aziz, Kuala Lumpur, 50300 Malaysia; 2 Drug Delivery System Excellence Centre, Department of Pharmaceutical Technology, Faculty of Pharmaceutical Sciences, Prince of Songkla University, Hat Yai, Songkhla, 90112 Thailand

**Keywords:** Pulmonary delivery, Biologics, Respiratory diseases, Asthma, COVID-19

## Abstract

**Abstract:**

The pulmonary route has gained significant attention as a drug delivery method, particularly for managing respiratory diseases. This approach provides several benefits, such as rapid therapeutic action, minimized systemic exposure, improved patient adherence, and the ability to deliver high drug concentrations directly to the lungs. Advances in inhalation devices, including pressurized metered-dose inhalers (pMDIs), dry powder inhalers (DPIs), and nebulizers, have established the pulmonary route as effective for administering both small-molecule drugs and complex biologics. Recent research has showcased the successful use of inhaled biologics such as monoclonal antibodies, nanobodies, and protein-based treatments in conditions like asthma, chronic obstructive pulmonary disease (COPD), pulmonary fibrosis, COVID-19, and respiratory syncytial virus (RSV). These treatments employ innovative mechanisms, such as muco-trapping and immune modulation, to optimize site-specific drug delivery and minimize systemic side effects. As technologies for pulmonary administration continue to evolve, they provide a non-invasive and highly promising platform for enhancing respiratory therapies and broadening the applications of biologics.

**Graphical abstract:**

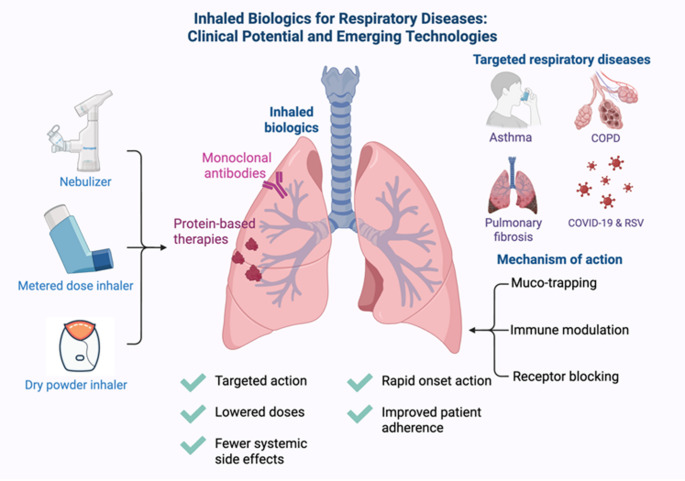

## Introduction

Since the global pandemic of SARS-CoV-2 arose, awareness of pulmonary diseases has emerged, reshaping priorities in respiratory health. Hence, pulmonary administrative delivery has garnered renewed attention for most pulmonary diseases. Inhaled therapeutics have been used for decades to treat lung diseases. It is the gold standard for the administration of low molecular-weight drugs [1]. However, the potential for expanding pulmonary delivery beyond traditional small molecules into the realm of complex biologics is now being seriously reconsidered.

Pulmonary administration has become one of the most favourable and non-invasive drug delivery techniques due to its ability to target lungs directly, reducing exposure of the used drugs to other locations [2]. Pulmonary therapeutic delivery enables administration of lower drug doses with reduced incidence of systemic adverse effects while also facilitates rapid onset of action for certain therapeutic agents [3]. Delivering therapeutics via inhalation requires navigating a highly complex biological landscape. For pulmonary delivery, the particle must cross several airway bifurcations.

While promising, there are a few setbacks in delivering therapeutics through inhalation. An example of the complexity of this system is the defence mechanism of the respiratory tract that has been established to prevent inhaled contaminants from entering the lungs and to remove or inactivate them after they have been deposited. Furthermore, an inhaling device or medium would be needed and used correctly to ensure ideal drug delivery [3]. The physical and immunological barriers in the lung are made up of mucus, the epithelial cell layer, surface fluid that is antimicrobial-rich, and neutralising immunoglobulins [4]. This barrier’s main function is to shield the airway from external hazards, but it also prevents effective drug administration. Despite these challenges, the unique physiology of the pulmonary system characterized by a vast surface area and proximity to the systemic circulation offers significant advantages for therapeutics that require rapid absorption and action, such as corticosteroids [5]. This biological “gateway” has driven increasing research interest in pulmonary delivery for more sophisticated agents, particularly biologics.

Biological therapeutics (biologics) cover a broad spectrum of products, including vaccines, blood-based substances, recombinant proteins, and somatic cells. They can be sourced from human, animal, or microbial origins and are frequently manufactured using advanced biotechnological processes. Cutting-edge treatments, such as gene-based or cell-based therapies, are at the forefront of biomedical innovation. In contrast to chemically synthesized drugs with distinct and fixed structures, many biologics are intricate blends that cannot be easily characterized. Biologics generally exhibit high specificity and lower toxicity levels compared to conventional drugs [6]. 

An example of a biological therapeutic is local-acting protein therapies such as Pulmozyme, a nebulized recombinant human DNAse [1] which has been commercialised for the treatment of cystic fibrosis (CF). Among protein-therapeutics, studies of antibodies-based therapeutics have also been increasing as antibodies are one of the prominent biological elements used for the targeted administration of drugs due to their framework’s stability, selectivity, and adaptability [7]. The ability of antibodies to accumulate in the target organ or tissue in a targeted, quantifiable manner regardless of the administration site and method is referred to as the use of antibodies in targeted drug delivery [8].

The goal of developing targeted pulmonary delivery systems is not merely to treat disease but to do so more safely by decreasing side effects and dosage requirements, efficiently by increasing plasma residence duration, and precisely by increasing the drug concentration at the target site, hence also improving biodistribution [9]. Nevertheless, while inhaled antibodies have shown promising preclinical and early clinical outcomes, no inhaled antibody therapies have yet been approved by major regulatory bodies such as the U.S. Food and Drug Administration (FDA) or European Medicines Agency (EMA). This gap between scientific promise and clinical approval highlights both the challenges and the untapped potential within this evolving field. Given the advancements in formulation strategies and delivery technologies, inhaled biologics are increasingly being explored as therapeutic options across a wide range of respiratory diseases. This review critically discusses recent developments in inhaled biologic therapies across various disease contexts, such as asthma, pulmonary fibrosis, chronic obstructive pulmonary disease (COPD), COVID-19 and respiratory syncytial virus (RSV). By evaluating the technologies and strategies specific to each condition, this review aims to highlight both the current progress and the future directions of inhaled biologic delivery.

### Pulmonary drug delivery as preferred route for respiratory diseases

The lungs are equipped with an extensive vascular system, holding about 500 mL of blood [10]. Their structure includes a vast epithelial surface spanning over 100 m² and a thin alveolar barrier less than 1 μm thick, enabling efficient gas exchange [11]. While the lungs have robust defence mechanisms, the pulmonary route for drug delivery remains highly promising and offers great potential to address unmet medical challenges.

The pulmonary drug delivery route ensures quick symptom relief due to the rapid onset of action, which is especially important during acute exacerbations [11]. In clinical settings, the rapid therapeutic onset provided by inhalation could be critically advantageous, especially in managing acute respiratory distress events where systemic drug administration might be too slow. Inhalation therapy requires a smaller dose compared to systemic administration, and it minimizes systemic exposure, significantly lowering the likelihood of side effects affecting the entire body [12].

A review comprising the administration of monoclonal antibodies via systemic administrations on COVID-19 has reported that the concentration of monoclonal antibodies (mAbs) in the lungs is typically reduced by 500–2000 folds than in the bloodstream [13–15]. Hence, inhalation delivery offers a more effective approach as it allows a significantly higher proportion of the administered mAbs to reach the target area directly within the airways [16]. This striking inefficiency of systemic administration strongly supports the argument for shifting towards direct inhalation methods to maximize therapeutic availability at the infection site. This approach ensures elevated and longer-lasting drug levels at the disease site, eliminating the need for large doses administered systemically.

Administering drugs directly to the lungs through inhalation can achieve both local and systemic therapeutic effects. Importantly, this dual-action capability underscores the versatility of pulmonary delivery, suggesting it could bridge the gap between targeted and systemic therapies, depending on the disease. Compared to other administration routes, pulmonary inhalation offers significant advantages such as a large absorption area with a thin alveolar epithelial cell membrane that facilitates rapid absorption and high permeability. This ensures rapid concentration of medication at the target site while keeping systemic drug levels low [17].

Considering how pulmonary route is particularly advantageous, when compared to oral delivery, albeit the convenience and non-invasive technique, it is often related to its poor bioavailability due to enzymatic degradation and first-pass metabolism in the liver, making it unsuitable for many biologics specifically the proteins and peptides [18]. In contrast to inhaled delivery route as it bypass hepatic first-pass metabolism primarily due to their route of administration, as they are directly delivered to the respiratory tract rather than absorbed via the gastrointestinal system. This results in reduced systemic metabolism and allows for higher local drug concentrations at the site of action, potentially improving therapeutic efficacy and also reduces systemic exposure [19].

If compared to intravenous and subcutaneous administrations, both are invasive in nature but has differences in their pharmacokinetic profiles. Intravenous delivery provides 100% bioavailability and rapid systemic distribution, but it requires clinical supervision and often fails to achieve sufficient drug concentration in the lungs, particularly for monoclonal antibodies targeting respiratory infections [20]. In contrast, subcutaneous injections, though more convenient than IV, are associated with delayed absorption and similarly result in limited pulmonary drug levels [21]. Hence, from a patient-centric perspective, the non-invasive nature of inhalation paired with the potential for dose reduction and fewer systemic side effects may enhance therapeutic adherence compared to injectable regimens [22]. These advantages make pulmonary delivery an attractive alternative for administering biologics.

The first traction of pulmonary drug delivery was in the 1950s specifically for asthma treatment and has become a widely accepted approach for managing respiratory conditions since. While initially developed for asthma management, the maturation of inhalation technologies now offers much broader therapeutic possibilities, extending well beyond classical respiratory conditions. Utilizing devices to spray or nebulize drug aerosols, this technique enables patients to breathe in medication, allowing faster achievement of peak lung concentration (Cmax) [23]. Animal studies have highlighted the superior efficiency of the inhalation drug delivery route as compared to intravenous or intraperitoneal administration for delivering mAbs targeting respiratory viruses like influenza and RSV. Furthermore, researchers observed that 1.7% of inhaled mAb 1212C2, a strong neutralizing antibody against SARS-CoV-2, reached the bronchoalveolar lavage fluid (BALF) of hamsters within 30 min of inhalation. In contrast, the intraperitoneal route resulted in less than 0.1% lung deposition of the same antibody [24]. These findings offer compelling preclinical validation that pulmonary delivery not only improves drug targeting efficiency but may also enable substantial dose reductions, minimizing costs and potential systemic side effects.

### Device considerations for inhaled biologics: bridging formulation and function

Effective pulmonary drug delivery depends not only on therapeutic agent, but also on the delivery method used. Various inhalation devices have been developed to optimize drug deposition in the lungs, particularly for patients with respiratory diseases such as asthma and COPD. Understanding the physiological requirements and device-speicific characteristics is crucial for achieving targeted delivery and therapeutic success. The following section outlines key considerations and the strengths and limitations of each delivery platform as shown in Table 1.

**Fig. 1:**
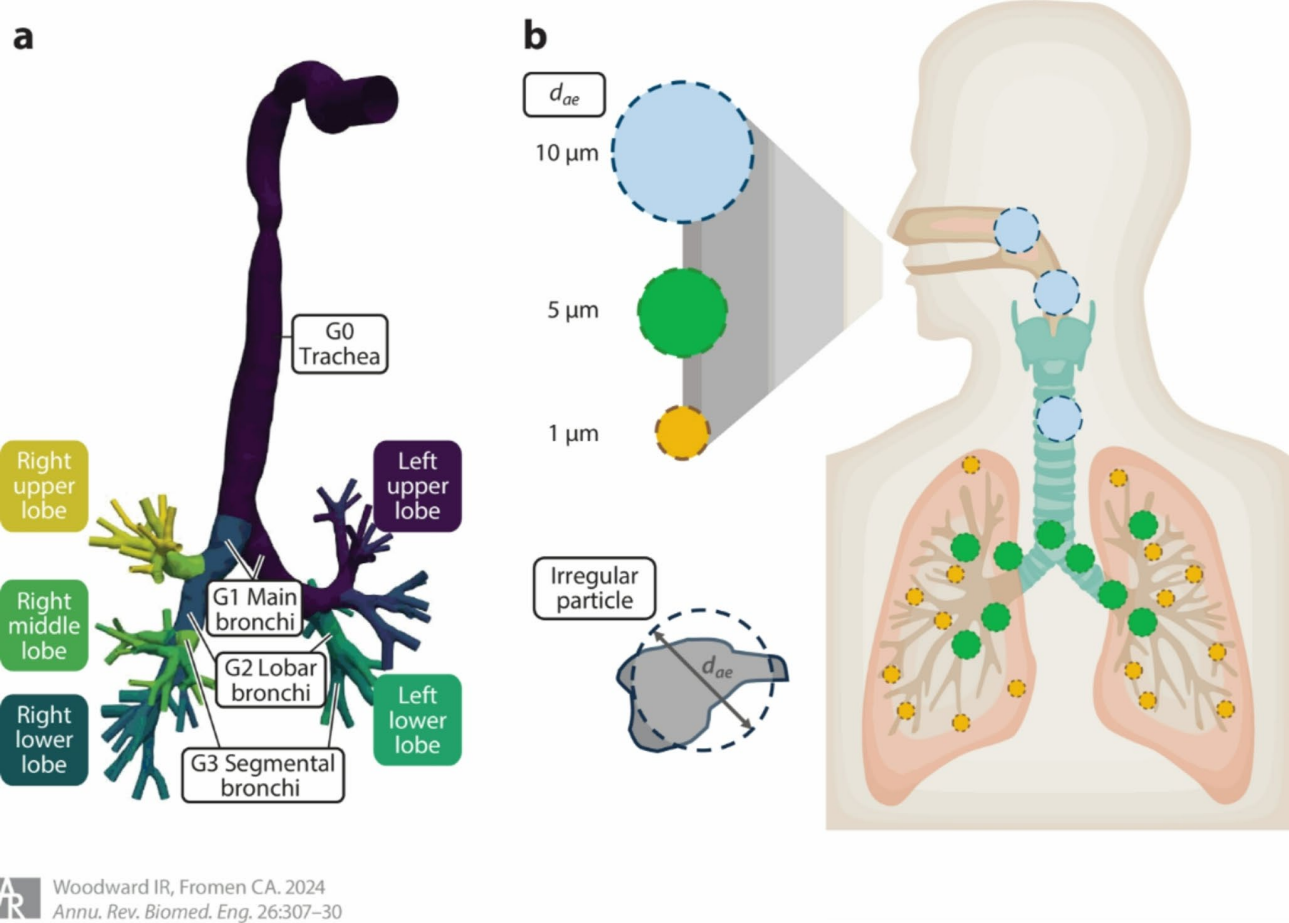
Illustration of size depositions of inhaled particles in the respiratory system. (**a**) Structure of the upper airway of the human lung. Mouth inlet is idealized, while the trachea and bronchi are obtained from a healthy adult male [31]. Generations 0–3 (G0–G3) are labeled, and representative generations are indicated for the lobar and segmental bronchi. (**b**) Diagram depicting the role of aerodynamic diameter (dae) in generational deposition within the lung, where ∼10-µm aerosols deposit in the oropharynx and trachea, ∼5-µm aerosols deposit in the upper conducting airways, and ∼1-µm aerosols reach the respiratory airways [28]. Illustration adapted from [32]

The physicochemical characteristics of aerosols, such as their shape, size, density, and hygroscopicity, play a crucial role in determining how inhaled particles are deposited within the lungs [25]. Among these factors, the aerodynamic diameter of particles is particularly significant for dictating their distribution across various regions of the respiratory system. In order to establish an effective pulmonary delivery, inhaled biologics must be able reach and remain in the intended region of the lung. This is largely determined by the aerodynamic diameter of the particles. Particles with diameters ranging from 1 to 5 μm have been found to deposit more effectively in the lungs, which in turn enhances their potential therapeutic efficacy [26, 27]. Particles smaller than 1 μm may be exhaled before deposition, while those larger than 5 μm tend to deposit in the oropharyngeal region, thereby failing to reach deeper lung tissues [28, 29]. This structure of size depositions of inhaled particles in the respiratory system is shown in Fig. 1 [30].

Once deposited, the pharmacokinetics of the therapeutic agent are influenced by its molecular size and biochemical properties. Inhaled agents must also overcome shifts in humidity, penetrate the airway lining, and bypass various cellular defence mechanisms. The pulmonary immune landscape includes resident immune cells that play essential roles in maintaining airspace integrity, clearing inhaled particles, and initiating localized immune responses. Among these, alveolar macrophages, CD11b + and CD103 + dendritic cells, and interstitial macrophages are of particular interest, as they are involved in numerous respiratory pathologies [33].

In addition to deposition, effective pulmonary delivery requires adequate residence time within the lungs [34]. The retention and clearance of inhaled biologics can vary significantly depends on their size and structure. Monoclonal antibodies, due to their large molecular weight (~ 150 kDa), often show prolonged retention in the lung interstitium but are more prone to enzymatic degradation or uptake by alveolar macrophages [35–37]. In contrast, nanobodies and peptides, owing to their smaller sizes exhibit improved tissue penetration but shorter half-lives [38], necessitating formulation strategies like PEGylation [37, 38] or encapsulation [38] to enhance stability and residence time.

**Fig. 2:**
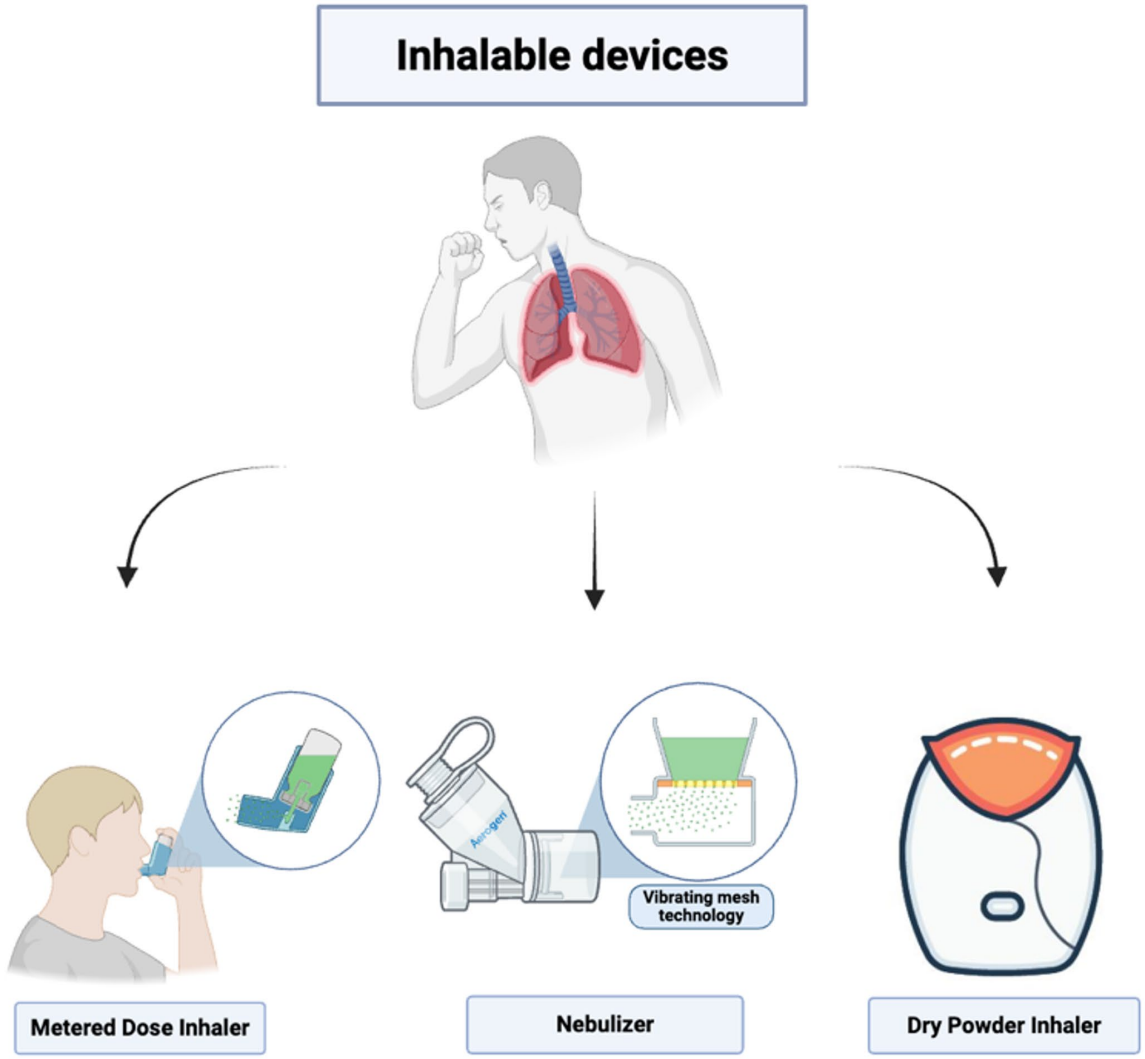
Types of inhalable devices for pulmonary delivery

Methods of pulmonary delivery include pressurized metered-dose inhalers (pMDIs), dry powders for inhalation (DPIs), and nebulizers [25] as shown in Fig. 2. Deciding between various options involves careful consideration of multiple aspects, such as the physicochemical characteristics of the drug, the simplicity of its application, the patient’s age, and the feasibility of manufacturing on an industrial scale [26]. Inhaler and the formulation design must also account for lung physiology, including mucociliary clearance, surface liquid thickness and alveolar macrophage activity, all of which influence particle retention and absorption [21]. Therapeutic aerosols must meet specific criteria regarding their aerodynamic particle size to be effective.

pMDIs include a pressurized canister containing a drug-propellant mixture that delivers medication in a fine mist upon actuation. It is often used with spacers to improve drug deposition. The advantages of using pMDIs are the portability, hand-held design, and the ability to deliver accurate doses with each actuation. However, reports have shown that excessive drug deposition was found in the oropharyngeal region, necessitating higher dosage levels to achieve therapeutic effects at the target site when using this device [26, 39]. DPIs have been manufactured to aid patients mainly suffering from asthma and COPD. Several manufacturers of DPIs produce active systems equipped with an external energy source to aid in the deagglomeration of the powder. These devices are particularly beneficial for individuals with reduced breathing capacity, such as children, critically ill patients, and those suffering from severe COPD or asthma [40]. The first researchers to introduce the use of DPI technology were Bell et al., where they demonstrated the ability of the device to dispense size-graded fractions of lactose, in the range of 4–400µ [41].

The emerging technology of the development of pressurized breath-activated inhalers (BAIs) aim to integrate the advantages of pMDIs and DPIs while addressing their shortcomings. For instance, devices such as Synchrobreathe™ operate by detecting low efforts of inhalation and synchronizing dose delivery with the breathing process, thereby eliminating the challenge of coordinating the two actions [42]. BAIs offer various benefits, including user-friendliness, efficient therapeutic delivery to the lungs, and greater patient preference. The future of medicine is set to prioritize tailored biological therapies and advancements in drug delivery systems. Smart inhalers integrated with sensors are also emerging, which track inhalation parameters and adherence, offering personalized dosing and real-time feedback for improved clinical outcomes [43, 44].

Nebulizers consist of three different categories which are jet nebulizers, ultrasonic nebulizers, and mesh nebulizers where the technology depends on the properties of drug solution into aerosols [26, 45]. The introduction of early nebulizers dates back to the mid-19th century, marking the initial step in aerosol medicine delivery. Later, between the 1930s and 1950s, advancements led to the creation of both electric nebulizers and hand-bulb models, further revolutionizing respiratory therapy [46]. Nebulizers operate effectively with tidal breathing and do not demand extensive patient training or cooperation. They are generally used for diseases that require high pulmonary doses and patients who are unable to achieve the necessary flow rate. As a result, they are particularly beneficial not only in emergencies but also for elderly and paediatric patients who may have cognitive or physical challenges [26, 45].

pMDIS are typically not suitable for delivering biologics due to the propellant-protein compatibility issues [30]. When administering drugs, the formulation would only content approximately 1% of the active drug with typically delivering less than 0.5 mg of medication per actuation [47, 48]. Studies have reported that only 10–20% of the drug dosage deposited by pMDIs is concentrated in the lungs [49]. DPIs on the other hand have better stability to deliver biologics with the ability to effectively aerosolize large masses (25–100 mg) of spray dried powder formulations [50, 51]. In comparison with nebulizers, they are capable of administering significantly large doses, often exceeding 100 mg, making them more suitable for therapies requiring higher drug loads [48]. Hence, more new developments of aerosol medications and most biologics involved requires nebulizer delivery.

The selection of an inhaler is also influenced by various patient-specific factors. This includes the patient’s inspiratory flow rate and volume, severity of the condition, presence of other health issues, and the patient’s ability to use the device [52]. It is also influenced by the properties of the developed formulation in consideration of the particle size and aerosol velocity generated by the device as well as the regimen complexity of inhalation therapy [53].

**Table 1:** Shows a comparison of pulmonary drug delivery devices

Device type	Mechanism/description	Advantages	Disadvantages	Suitability range	References
Pressurized metered-dose inhalers (pMDIs)	Propellant-driven canister releases aerosol upon actuation. Often used with spacers.	-Portable and convenient -Delivers accurate doses -Widely available	-Requires hand-breath coordination -High oropharyngeal deposition -Propellant-protein compatibility issues	Adults and adolescents with good inhalation coordination	[26, 30, 39]
Dry powder inhalers (DPIs)	Breath-actuated, releases powder formulation. Some use external energy for deagglomeration	-Suitable for nanobodies and peptides with stabilizers -Propellants are not needed, stable dry formulation -Breath-activated -Useful in asthma and COPD	-Requires strong inspiratory effort -Sensitive to humidity -Less effective in severe airflow limitation -Sheer stress may denature proteins	Asthma and/or COPD patients with sufficient inspiratory capacity	[30, 32, 40, 41]
Breath-activated inhalers (BAIs)	Combines pMDI and DPI benefits, dose released automatically upon inhalation	-Eliminates coordination need -Higher user-friendliness -Preferred by many patients	-Less availability -Higher cost -Fewer formulations	Children, elderly or cognitively impaired patients	[42]
Nebulizers	Converts liquid formulation to aerosol for inhalation, used during tidal breathing	-High lung deposition -Mesh type, gentle on proteins -Minimal coordination required -Suitable for high doses -Versatile for biologics	-Bulky -Longer treatment times -Requires cleaning and maintenance	Paediatric, geriatric or hospitalized patients	[26, 30, 45, 46]

### Inhaled biologics across respiratory pathologies: current strategies and emerging evidence

When delivering biologics specifically proteins, a main concern is protein degradation during administration. When proteins lack stability in their solution form, they can be enhanced with stabilizing agents before undergoing freeze-drying or spray-drying processes for preservation [54, 55]. These approaches allow the proteins to be reconstituted effectively, making them suitable for delivery through nebulizers. Studies have shown that aerosols produced using mesh nebulizers specifically the vibrating mesh technology significantly minimize protein degradation compared to jet or ultrasonic nebulizers, which rely on heating elements [14, 56]. While traditional jet nebulizers deliver medication with an efficiency of approximately 10%, newer vibrating mesh nebulizers (VMNs) surpass 60% efficiency, tackling challenges often seen in dry powder formulations for proteins such as hygroscopic growth and protein aggregation. Additionally, these devices eliminate the need for synchronized breathing techniques that are frequently required with dry powder inhalers or metered dose inhalers, which can pose difficulties for both elderly and paediatric users [14, 15].

Limitation of MDI that may occur to delivering biologics are such as the hydrophilic nature of most biologics and poorly soluble in the non-polar hydrofluoroalkane (HFA) propellants used in MDIs. This poor solubility limits the formulation possibilities and the dose range that can be delivered per actuation [57]. Besides that, MDIs often result in high oropharyngeal deposition and require precise coordination between actuation and inhalation. This can lead to suboptimal lung deposition, especially in patients who have difficulty with inhaler techniques [58]. Hence, nebulizers are highly favoured for administering proteins due to the simplicity of their formulations and the compatibility with soluble proteins, which require minimal use of additives.

Given the continuous advancements in formulation techniques and delivery technologies, inhaled biologics are emerging as promising therapeutic options for a wide range of respiratory disorders. This section provides a comprehensive analysis of recent developments in inhaled biologic therapies, focusing on diseases such as asthma, pulmonary fibrosis, COPD, COVID-19, and respiratory syncytial virus (RSV). By evaluating the specific delivery strategies and technologies for each condition, this review aims to highlight both current progress and future directions in the field. A summary of the key findings discussed is presented in Table 2.

**Table 2:** Shows a summary of the key findings discussed in section below

Respiratory disease	Biologics involved	Type of biologics	Mechanism of action	Development status	References
Asthma	Ecleralimab	mAb fragment	Anti-TSLP therapy	Phase II clinical trial	[16, 59]
	CDP7766	mAb fragment	Prevents IL-13 from binding to its receptor, IL-13Rα1	Preclinical animal-model study	[60]
Pulmonary fibrosis	OM-85	Bacterial lysate	Promote a Th1-biased immune environment by increasing interferon-gamma (IFN-γ) and reducing interleukin-4 (IL-4)	Preclinical animal-model study	[61]
	PRS-220	Protein	Blocks CCN2, preventing the over-activation of fibroblasts and the excessive deposition of extracellular matrix components	Preclinical ex-vivo human study	[62]
	Lung spheroid cell secretomes or exosomes	Biologic mixture or nanoparticle-based biologic	Modulates fibrosis, inflammation, and tissue repair processes	Preclinical animal-model study	[63]
COVID-19	Pittsburgh inhalable Nanobody 21 (PiN-21)	Single-domain antibody fragment	Bioengineered into a trimeric form for higher affinity towards SARS-CoV-2	Preclinical animal-model study	[64]
	SNG001	Protein (recombinant cytokine)	Increases high concentrations of interferon-β in the lungs resulting in a robust local antiviral response	Phase II clinical trial	[65]
	HH-120	Multivalent, recombinant fusion protein	High binding affinity to the viral S protein, increasing ability of viral neutralization	Preclinical animal-model study	[66]
	IN-006	mAb	Immobilizes SARS-CoV within the airway mucus, preventing the trapped virions from diffusing through the mucus to reach and infect host cells (Muco-trapping)	Preclinical animal-model study	[67]
Chronic Obstructive Pulmonary Disease (COPD)	Progesterone (P4)	Hormone	Modulate pathological balance in the lungs with significant anti-inflammatory and antioxidant properties	Preclinical animal-model study	[68, 69]
	Patchouli essential oil (PEO)	Plant extract	Reduces inflammatory cell infiltration and decreases the levels of pro-inflammatory cytokines such as IL-6 and TNF-α	Preclinical animal-model study	[70]
	Immunoantimicrobial nanoparticles (IMAMs)	Peptides	Reduces airway bacterial burden, decreases pro-inflammatory cytokine levels and suppress TLR9 signalling	Preclinical animal-model study	[71]
Respiratory syncytial virus (RSV)	Mota-MT	mAb	Muco-trapping therapy, neutralizing monoclonal antibody targeting the RSV F	Preclinical animal-model study	[72]
	ALX-0171	Nanobody	Targets the F protein of RSV to block the virus from entering host cells	Phase I/IIa clinical trial	[73–75]

#### Inhaled biologics for asthma: direct targeting of TSLP and IL-13 pathways

Asthma is a common and persistent respiratory illness that impacts millions worldwide. Its symptoms include wheezing, breathing difficulties, chest tightness, and coughing. The condition has a far-reaching effect, with approximately 262 million people affected in 2019, predominantly children, resulting in significant mortality and economic burdens. Recent data from the Global Initiative for Asthma (GINA) 2024 Report indicates the global asthma population has now approached 300 million individuals [76].

The pharmacological management of asthma is divided into several categories such as rescue therapies, controller treatments, and, for severe cases, add-on options. Reliever medications play a key role in addressing acute symptoms, while controller therapies are designed to minimize airway inflammation. For patients with severe asthma, particularly those with an eosinophilic phenotype, biologic treatments are often recommended. These patients typically continue to experience symptoms and frequent exacerbations despite using high-dose inhaled corticosteroids (ICS) combined with long-acting beta-agonists (LABA). Current biologic therapies target molecules such as immunoglobulin E (IgE), IL-4, IL-5, IL-13, and thymic stromal lymphopoietin [77]. The EMA and the U.S. FDA have approved six biological treatments, including omalizumab (anti-IgE), benralizumab, mepolizumab, reslizumab (anti-IL5/IL5R), dupilumab (anti-IL-4Rα/IL-13), and tezepelumab (anti-TSLP) [78–82]. While systemic biologics have made significant strides, it is notable that inhaled options could offer a transformative step forward by delivering treatment directly to the site of inflammation with reduced systemic exposure. However, all approved biological therapies are administered via systemic routes, including intravenous and subcutaneous methods.

Systemic administration of biologics is associated with key limitations such as low pulmonary bioavailability due to distribution dynamics, reducing local efficacy [83]. Moreover, systemic exposure increases the risk of adverse effects including hypersensitivity reactions, generalized immunosuppression and potential toxicity. Biologics delivered systemically may also exhibit delayed onset due to longer absorption and distribution phases [84]. These limitations underscore the need for direct, localized delivery methods that can improve drug concentrations in lung tissue while minimizing systemic exposure. Such approaches may also enhance patient adherence by reducing the invasiveness and complexity of treatment regimens.

**Fig. 3:**
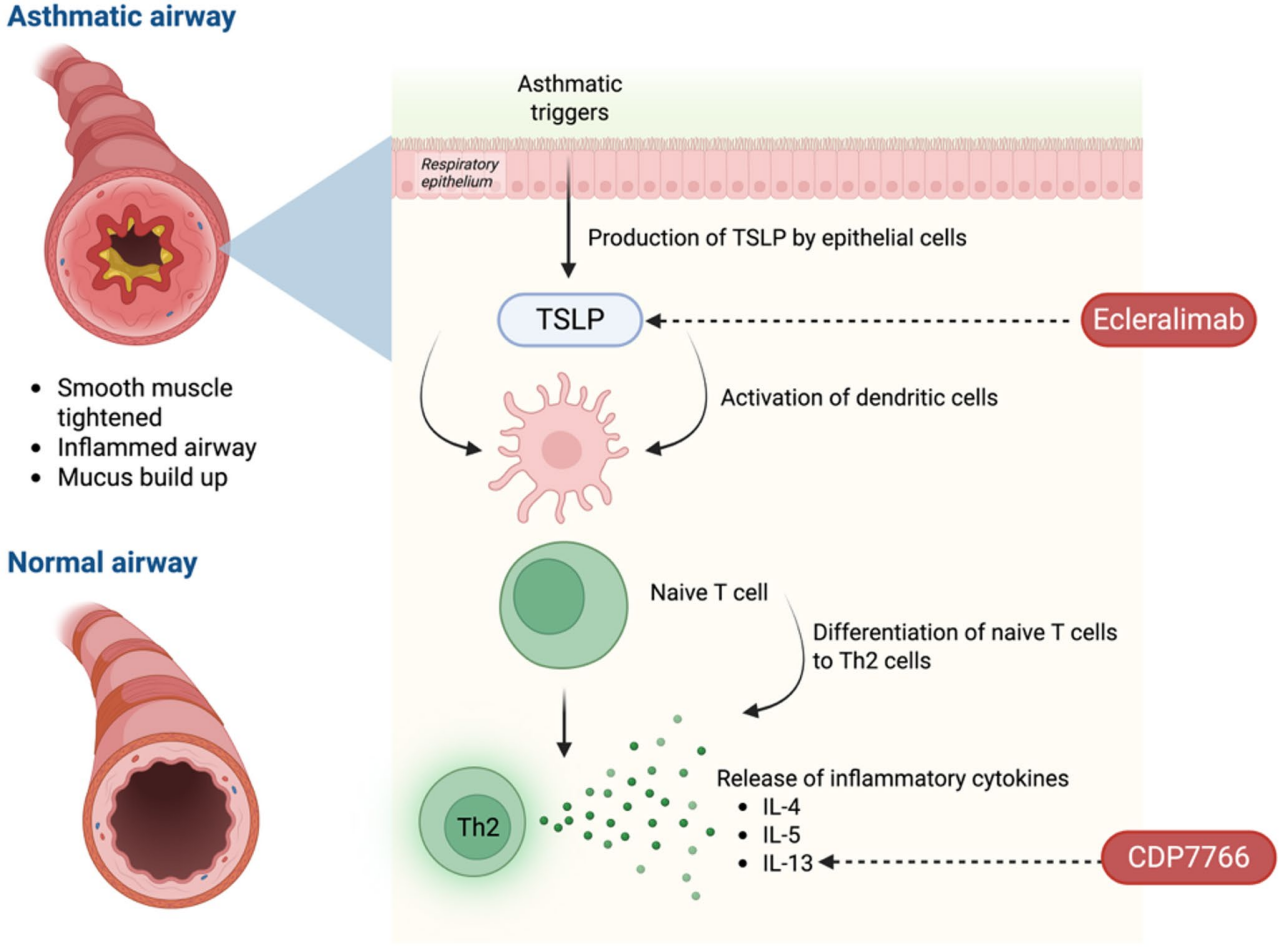
An illustration of asthmatic airways and the production of key cytokines involved. Ecleralimab targets the TSLP involved in anti-TSLP therapy while CDP7766 targets the IL-13 to effectively reduce IL-13 activity. (Created in Biorender.com)

Thymic stromal lymphopoietin (TSLP), a cytokine produced by epithelial cells, plays a critical upstream role in the development of asthma. TSLP is involved in activating dendritic cells, which leads to the differentiation of naïve T cells into Th2 cells. These Th2 cells then produce cytokines such as IL-4, IL-5, and IL-13 (Fig. 3) [16]. Research suggests that inhibiting TSLP with the monoclonal antibody Tezepelumab which is currently the only approved add-on treatment could benefit a wide range of asthma patients [85], however, limited to systemic administration methods, necessitating regular injections. Reliance on systemic administration may hinder the full therapeutic potential of TSLP-targeting agents due to the difference in dosing schedules and limited drug targeting, inhaled biologic therapies targeting TSLP could offer a practical alternative by improving local pharmacodynamics and reducing systemic side effects.

A recent study reported by Gauvreau et al., has developed ecleralimab as the pioneering inhaled anti-TSLP therapy aimed at treating asthma [16, 59]. Phase II clinical trial was done to evaluate its efficacy and safety which involves subjects with mild atopic asthma to proceed to development in severe asthmatic subjects. In this study, ecleralimab, a potent neutralizing antibody fragment belonging to the fragment antigen-binding (Fab) group and falls under the IgG1/λ isotype subclass, specifically targets human TSLP and is formulated as PulmoSol™, a powder designed for administration through a dry powder inhaler (DPI) device, using hard capsules. This antibody fragment comprises the antigen-binding components, including one constant and one variable domain from both the heavy and light chains, but it does not include the Fc region. The smaller size of Fab fragments (46.6 kDa compared to full antibodies at ~ 150 kDa) allows better penetration of lung tissue. With a molecular weight of 46.6 kDa, it is notably smaller compared to the approximately 150 kDa size of a complete antibody [16]. With no Fc region, Fab fragments are small enough to penetrate airway tissue, while maintaining antigen-binding specificity. Although a lack of Fc region means a shorter serum half-life, this is ideal for inhalation therapies as it gives a more localized therapeutic effect, targeting the airways in asthmatic patients and a faster elimination process reducing its side effects [16, 60]. Results showed that ecleralimab effectively reduced bronchoconstriction and inflammation in the airways triggered by allergens. Moreover, it demonstrated a favourable safety profile in individuals with mild atopic asthma [59].

Monoclonal antibodies (mAbs), a common biological agent are immunoglobulins with a high degree of specificity for the particular antigen or molecule for which they were developed [86]. The ground-breaking work of Kohler and Milstein, who successfully generated the first mAbs from a single B-cell hybridoma cell line using the hybridoma technique [9]. Notably, the FDA approved the first therapeutic mAbs, muromonab-CD3, which is adapted to transplantation procedures [7]. mAbs play a pivotal role in the treatment of a wide spectrum of conditions, including cancer therapy, autoimmune diseases, prevention of organ transplant rejection, and targeted drug delivery [9]. The increasing interest in inhaled mAbs reflects a broader trend in respiratory therapeutics aimed at achieving localized action while minimizing systemic risks.

IL-13, a cytokine significantly involved in the pathogenesis of severe allergic asthma, has also emerged as a promising target for inhalation-based therapies [87]. Research conducted by Lightwood et al. has developed CDP7766, a monoclonal fragment antigen-binding (Fab) antibody with high potency and is biophysically stable with strong specificity and affinity for IL-13. This functions by preventing IL-13 from binding to its receptor, IL-13Rα1, thereby inhibiting the formation of the high-affinity IL-13:IL-13Rα1:IL-4Rα signalling complex. In preclinical trials, inhaled CDP7766 using an ultrasonic nebulizer effectively reduced IL-13 activity, as well as the associated upregulation of cytokines and chemokines, and mitigated allergen-induced increases in pulmonary resistance. Importantly, no adverse effects from local irritation were observed [60]. These findings provide a strong basis for advancing the investigation of inhaled CDP7766 as a potential therapy for allergic asthma in humans. However, translation to clinical use requires further investigation in large-scale trials to confirm efficacy, safety and optimal delivery strategies. Nonetheless, the transition from systemic to inhaled biologics will require careful evaluation to ensure that the pharmacokinetics and bioactivity of the antibodies are not compromised. Hence, based on these findings, inhaled biologics offer a highly promising but still evolving frontier that warrants deeper exploration in both clinical and real-world settings.

#### Localized treatment strategies for pulmonary fibrosis: anticalins, exosomes, and immunomodulation

**Fig. 4:**
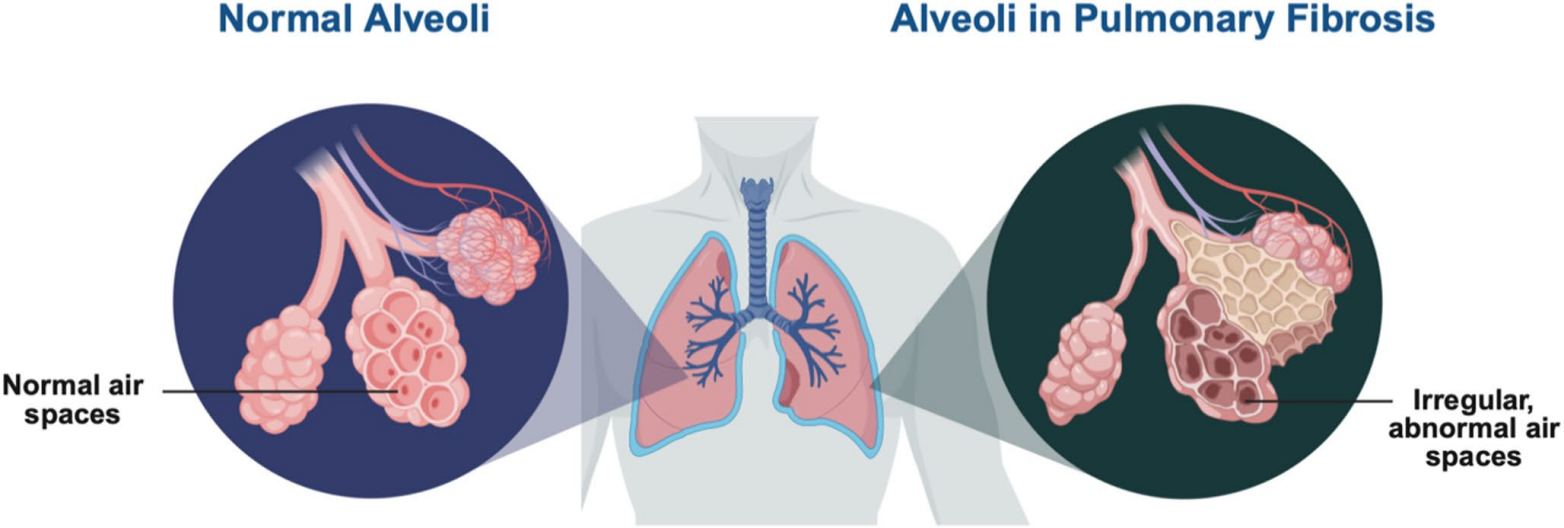
An illustration of normal alveoli and alveoli in pulmonary fibrosis. (Created in Biorender)

Idiopathic Pulmonary Fibrosis (IPF) is a chronic interstitial lung condition, primarily characterized by scarring of the alveolar-capillary interface (Fig. 4). Excessive apoptosis and injury of the alveolar epithelium caused by increased profibrotic factors disrupt the normal gas exchange and ultimately culminate in respiratory failure [88, 89]. Despite extensive research, the main causative factor of IPF remains obscure. IPF is often diagnosed in older adults, with very poor prognosis, in which patients typically survive only 3 to 5 years following diagnosis. In contrast to other inflammatory lung diseases, IPF shows minimal to non-effective towards anti-inflammatory treatments, and in some cases, treatments lead to exacerbation of the condition [90]. Currently, the approved therapeutic regimes for IPF are nintedanib, a kinase inhibitor, and pirfenidone, which aim to inhibit extracellular matrix deposition and slow down the fibrotic processes [91–93]. However, these agents do not reverse fibrosis or even show any sign of cure. Among interstitial lung diseases, IPF garners particular clinical attention due to its severe progression, poor treatment outcomes, and high mortality rate [94].

Given the limited efficacy of existing treatments, research is moving towards understanding the underlying mechanisms of fibrosis. It is hypothesized that the balance between T-helper subtypes, Th1 and Th2, plays a pivotal role in lung inflammation and fibrosis. Under normal physiological lung conditions, equal levels of Th1 and Th2 are crucial in maintaining cell immunity. In IPF, secretion of Th1/Th2 may suppress or increase the progression of pulmonary fibrosis, due to their regulatory roles [95, 96].

A recent study by Yu et al. utilized OM-85, which comprises bacterial lysates from bacterial pathogens, and evaluated its inhibitory effect in a mice pulmonary fibrosis model [61]. In this study, bleomycin-induced pulmonary fibrosis mice were given via aerosol exposure of OM-85 on days 42, 44, 46, 49, 51, and 53. Mice treated with aerosolized OM-85 exhibited significantly reduced fibrosis, as observed by decreased collagen accumulation, lower hydroxyproline content, and reduced inflammatory infiltration. Upon histopathological assessment, notable lung architecture was preserved and reduced Ashcroft scores were observed. OM-85 was shown to promote a Th1-biased immune environment by increasing interferon-gamma (IFN-γ) and reducing interleukin-4 (IL-4), thereby potentially regulating the fibrogenic Th2 shift induced by lung injury. OM-85 suppressed the expression of TGF-β1, Notch1, and Hes1, which are the key signalling molecules involved in fibrogenesis and fibroblast activation. Importantly, inhalation treatment of OM-85 reversed the Th2-skewed immune shift. These findings highlighted OM-85 as a promising inhaled immunomodulatory therapy as a potential treatment for IPF [61]. Taken together, the dual mechanism, preclinical efficacy, and pulmonary delivery characteristics proved OM-85 as a strong candidate for a novel and localized therapeutic strategy for IPF management.

Other than treatments targeting Th1/Th2 balance in IPF, inhaled therapies targeting cellular communication network factor 2 (CCN2) have also emerged as a promising approach for treating pulmonary fibrosis. CCN2, also known as connective tissue growth factor (CTGF), holds an important role in fibrotic tissue remodelling, wherein, its over-expression in lung tissues directly contributes to the progression of IPF. PRS-220, an inhaled Anticalin^®^ protein, is designed to specifically target CCN2 in the lungs, offering a novel approach to treating IPF and other fibrotic lung diseases. By blocking CCN2, PRS-220 prevents the over-activation of fibroblasts and the excessive deposition of extracellular matrix components. In preclinical concept studies by Neiens et al., pulmonary delivery of PRS-220 enables more efficient penetration to the site of action, the fibrotic lung tissue, when compared to systemic antibody [62]. With this localized delivery of PRS-220, a high concentration of PRS-220 can be reached with very minimal systemic exposure, thereby maximizing its therapeutic efficacy. PRS-220 was shown to accumulate in fibrotic lung tissue after inhalation, leading to a reduction in CCN2 levels and attenuation of the fibrotic processes.

These studies have shown that pulmonary delivery of treatments offers site-specific targeting, allowing effective treatment for pulmonary fibrosis when compared to systemic delivery. PRS-220, when delivered via inhalation, significantly reduced collagen deposition, fibrosis-associated proteins like α-SMA, collagen I/III, and fibronectin, and improved lung architecture. Overall, the preclinical profile of PRS-220 supports the hypothesis that local pulmonary delivery of Anticalin^®^ proteins is a promising therapeutic approach for pulmonary fibrosis.

In another preclinical study by Dinh et al., lung spheroid cell secretomes and exosomes were studied as a therapeutic approach to be delivered to the lungs of a fibrotic mice model [63]. Lung spheroid cells secrete a variety of regenerative factors, and their secretome, along with exosomes, was shown to have a significant impact on modulating fibrosis, inflammation, and tissue repair processes. Pulmonary fibrosis was induced in C57BL/6 mice using bleomycin, and the animals were then treated with inhaled lung spheroid cell secretomes or exosomes. Histological analysis revealed that the treated mice exhibited significant reductions in fibrotic lesions and collagen deposition when compared to the untreated group. In addition to fibrosis reduction, the inhaled treatments led to a decrease in the expression of fibrosis-related markers, such as collagen I/III, α-SMA, and fibronectin, within the lung tissue. Furthermore, the treatments appeared to have a regenerative effect, promoting epithelial repair and enhancing alveolar architecture. Increased epithelial proliferation was observed, which is a sign of tissue repair, that may help counteract the damage caused by fibrosis. The inhaled exosomes and secretomes regulate the immune levels in the lungs to a more balanced immune response. Specifically, the treatments increased the levels of Th1 cytokines such as IFN-γ, while decreasing Th2 cytokines like IL-4. Following treatment of inhaled exosomes and secretomes, impaired lung function significantly showed improvement. Overall, this study highlighted the therapeutic potential of inhaled lung spheroid cell secretomes and exosomes as a promising strategy for treating pulmonary fibrosis.

#### Inhaled biologics for COVID-19: nanobodies, interferons, and muco-trapping therapies

Biologics such as nanobodies, due to their lightweight molecular structure (approximately 15 kDa) and stable biophysical characteristics have emerged as significant potential therapeutics for nebulization in biological therapies. An ultrapotent homotrimer construct, Pittsburgh inhalable Nanobody 21 (PiN-21), has shown promising antiviral results as SARS-CoV-2 infectivity was efficiently blocked at below 0.1 ng/ml in vitro [64]. Researchers then continued to study the antiviral potential of PiN-21 in Syrian hamsters. Results showed that the aerosol administration using a vibrating mesh nebulizer of PiN-21 promotes effective distribution across the respiratory tract while enabling a reduced dosage of 0.2 mg/kg. This inhalation therapy not only expedites recovery from infection-induced weight loss in animals but also lowers lung viral titers by six logs, significantly alleviating lung damage and effectively preventing viral pneumonia [97]. The ability of PiN-21 to achieve such profound antiviral effects at ultra-low doses is particularly impressive and highlights the potential of nanobodies as next-generation inhalable antivirals.

Another formulation designed for nebulized inhalation is SNG001, a recombinant interferon beta formulation. In previous clinical trials, SNG001 has shown the ability to enhance lung antiviral defences in patients with asthma and/or COPD with or without respiratory viral infections [98, 99]. The inhalation delivery method was preferred to achieve high concentrations of interferon-β in the lungs, promoting a strong localized antiviral response while minimizing systemic exposure, which is linked to flu-like symptoms [98, 100]. In individuals with COVID-19, a diminished level of interferon-β was observed especially in the elderly or those with chronic respiratory illnesses. Combining the promising results of SNG001 on respiratory illness previously stated as well as the suppression of interferon-β by SARS-CoV-2, Monk et al., [65] reported a phase II clinical trial on inhalation via nebuliser of SNG001 on COVID-19 patients. It was shown to be well-tolerated with no deaths as compared to three deaths for the placebo-treated group and had greater odds of improvement on the OSCI (WHO Ordinal Scale for Clinical Improvement) scale [65].

Angiotensin-converting enzyme 2 receptor (ACE2) plays a significant role in the entry of SARS-CoV-2 into the host cells. The lungs have an abundance of ACE2 receptors making them the main target organ for COVID-19 as the S protein of SARS-CoV-2 binds to the ACE2 receptor for viral entry [101–103]. Proteins sourced from the extracellular domain of human ACE2 (hACE2) can act as decoys to block viruses from attaching to host cells. These soluble hACE2 proteins exhibit inherent resistance to viral mutational escape, making them a robust tool in countering viral infections. The development of HH-120, a soluble hACE2 molecule engineered into an IgM-like multivalent Fc-fusion protein by Liu et al., highlights significant advancements in SARS-CoV-2 therapeutics [66]. HH-120 demonstrates exceptional binding affinity to the viral S protein (> 1 × 10 − 12 M) and achieves substantial neutralization activity whereby approximately 88–265 times greater compared to hACE2-hIgG1, a bi-valent ACE2 tagged with human IgG1 Fc when tested against the ancestral strain (IVDC-QD-11-2P2) in Vero cells [66]. In vivo studies using golden Syrian hamsters showed that inhaled aerosolized HH-120 via VMN for early treatment resulted in potent antiviral effects, reducing lung pathology scores and leading to ~ 3 log reductions in viral loads [66]. Hence, this formulation has paved the way for clinical development as a reliable, convenient treatment against SARS-CoV-2 variants and other emerging ACE2-utilizing coronaviruses.

**Fig. 5:**
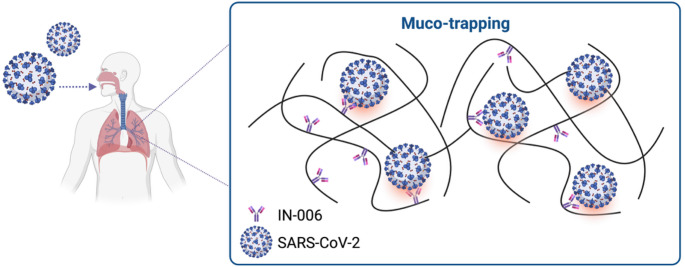
An illustration of muco-trapping mechanism illustrating SARS-CoV-2 moves freely in mucin matrix whereas Fc domain of antibody creates crosslinks with the mucin while Fab domain binds to virus. (Created in Biorender.com)

An emerging yet under-recognized mechanism during pulmonary delivery is muco-trapping which involves the antibodies to combat viral infections on mucosal surfaces. This mechanism happens due to the presence of mucin, the predominant component of mucus covering the respiratory tract’s luminal surface allowing viruses to move freely within its network of fibres [13, 73, 104]. Studies suggest that the Fc domain of IgG can create crosslinks with mucins, while the Fab domain binds specifically to viral antigens, effectively trapping the virus within the mucin structure (Fig. 5**).** This process aids in the clearance of viruses via mucus elimination pathways and prevents their entry into host cells during the early stages of infection [73]. An application of this mechanism is reported using IN-006 [67], a reformulated for nebulized delivery of regdanvimab, one of the approved neutralizing mAbs targeting SARS-CoV-2. IN-006 has demonstrated muco-trapping capabilities in human airway mucus where when nebulized using VMN to rats, the mAb level in the lungs was 100-fold greater as compared to the serum without compromising its activity. Substantial results have shown that after administration of IN-006, SARS-CoV was immobilized within the airway mucus, preventing the trapped virions from diffusing through the mucus to reach and infect host cells. These trapped virions will then be rapidly cleared from the respiratory tract through mucociliary action or cough-induced mucus elimination [67]. The muco-trapping mechanism employed by inhaled antibodies like IN-006 offers a fascinating and underexplored method for enhancing mucosal immunity, potentially adding a critical layer of defence at the earliest stages of infection.

#### Innovative pulmonary delivery for copd: hormones, plant extracts, and antimicrobial nanoparticles

Chronic Obstructive Pulmonary Disease (COPD) is a chronic inflammatory lung disease characterized by deterioration of lung function due to abnormal inflammatory reactions triggered by cigarettes or any airborne toxins. COPD is often associated with high morbidity and mortality, and is the third leading cause of death globally [105]. Despite the advancement of the use of inhaled bronchodilators and corticosteroids, these treatments only address symptoms rather than curing the disease progression, highlighting the urgent need for novel therapeutic approaches that can regulate and cure the pathogenesis of COPD [106–108].

Recent evidence has highlighted the promising role of hormones in immune regulation, particularly progesterone (P4), a steroid hormone important in reproductive health. Beyond its endocrine functions, P4 exhibits significant anti-inflammatory and antioxidant properties [68, 69]. A 2025 preclinical study by Xie et al., explored the therapeutic potential of progesterone in a murine model of cigarette smoke-induced COPD [109]. BALB/c mice were subjected to chronic smoke exposure and were given intranasal treatment of P4 to establish localized pulmonary administration. Pulmonary delivery of P4 was intended to maximize drug deposition in the lungs, limit systemic side effects, and directly target airway inflammation and oxidative stress of the COPD-induced mice model. Treatment with P4 revealed notable therapeutic outcomes. Histopathological examination revealed preserved alveolar architecture in the lungs, reduced neutrophilic infiltration, and reduced emphysematous changes in P4-treated animals. Inflammatory profiling of bronchoalveolar lavage fluid (BALF) indicated a significant reduction of pro-inflammatory cytokines such as TNF-α and IL-6, along with decreased activity of matrix metalloproteinase-9 (MMP-9), a key enzyme involved in extracellular matrix degradation. Moreover, P4 restored the oxidative balance by enhancing levels of endogenous antioxidants, superoxide dismutase (SOD), and catalase, while concurrently decreasing malondialdehyde (MDA), a lipid peroxidation marker. Altogether, this study has proven and revealed progesterone as a promising candidate for inhalation-based therapy in COPD, with its ability to modulate pathological balance in the lungs, other than the traditional anti-inflammatory treatments that only target disease symptoms.

Other than the pulmonary delivery of hormones as a therapeutic approach against COPD, growing interest in plant-derived compounds with bioactive properties as an alternative intervention. One such candidate is patchouli essential oil (PEO), extracted from *Pogostemon cablin*, traditionally used in herbal medicine for its anti-inflammatory, antimicrobial, and antioxidant effects. A recent experimental study by Zhang et al. evaluated the efficacy of inhaled PEO in a murine model of cigarette smoke-induced COPD [70]. Mice were exposed to chronic cigarette smoke to establish a COPD-mice model and were subsequently treated with PEO via inhalation to assess its direct impact on lung inflammation and tissue remodelling. Inhalation treatment of PEO produced significant improvements in the pulmonary area. PEO-treated mice showed significant reductions in inflammatory cell infiltration within bronchoalveolar lavage fluid (BALF), together with decreased levels of pro-inflammatory cytokines such as IL-6 and TNF-α. Histological analysis also revealed preservation of alveolar architecture and reduced signs of airway remodelling compared to untreated COPD mice controls. It was believed that PEO regulated the inflammatory pathways by modulating pathways involved in oxidative stress and cytokine production. Taken together, the study highlighted the novel, effective approach of inhaled patchouli essential oil as a natural, lung-targeted anti-inflammatory agent.

A recent inhalation study investigated the therapeutic potential of immunoantimicrobial nanoparticles (IMAMs) pulmonary delivery in the treatment of infection-driven COPD exacerbations [71]. The IMAMs consisted of negatively charged porous silica nanoparticles encapsulating ceftazidime and pH-responsive antimicrobial peptides (AMPs). These were specifically engineered to overcome the physical barriers of the respiratory tract, including mucus and bacterial biofilms, while maximizing therapeutic action at the disease site. In a murine model of COPD-like lung infection, the IMAMs were administered via nebulization, allowing direct deposition into the lower airways and ensuring a high localized concentration of therapeutic agents. The nanoparticle surface charge and aerodynamic profile allowed for efficient mucus penetration and deep lung delivery. Upon reaching the acidic microenvironment of infected tissue, the AMP component underwent structural transformation, enhancing biofilm disruption and promoting bacterial eradication at the alveolar level. Pulmonary delivery of the IMAMs resulted in significant therapeutic benefits, including reduced airway bacterial burden, decreased pro-inflammatory cytokine levels in bronchoalveolar lavage fluid (BALF), and suppression of TLR9 signaling. Histological analysis confirmed reduced lung inflammation and damage in the IMAM-treated group. This inhalation approach not only ensured targeted delivery and retention within the lungs but also minimized systemic exposure and off-target effects. Strong preclinical evidence from the study supports the potential of nebulized IMAMs as a dual-function inhalation therapy for COPD [71]. By effectively navigating pulmonary barriers and delivering both antimicrobial and immunomodulatory payloads directly to the lungs, this strategy offers a promising avenue for disease modification in COPD beyond traditional symptomatic treatment.

To conclude, these recent preclinical studies highlight the growing potential of pulmonary delivery therapies as a localized and multifaceted approach for the management of COPD. Whether through the hormone anti-inflammatory and antioxidant characteristics, the bioactivity properties of essential oil, or immunoantimicrobial nanoparticles, each approach demonstrated that targeted pulmonary delivery not only enhances drug concentration at the disease site but also reduces overall systemic. These findings signal a paradigm shift from symptomatic control towards disease-control mechanisms.

#### RSV intervention via inhaled biologics: muco-trapping antibodies and nanobody-based antivirals

For respiratory infections, inhaling anti-infectious antibodies may be more effective than the traditional intravenous (IV) injection, as it allows for direct targeting of the infection site and enhances the therapeutic index of the antibodies.

Respiratory syncytial virus (RSV) is the leading cause of lower respiratory tract infections (LRTIs) in young children and significantly impacts morbidity and mortality among the immunocompromised and elderly [110, 111]. Worldwide, RSV is responsible for around 35 to 40 million cases of acute LRTIs annually in children under the age of 5. In the United States, approximately 2 million children under 5 are affected by RSV infections each year, necessitating medical care, and about 2–3% of these cases (around 40,000–60,000) result in hospitalization [72, 112]. Given the substantial global burden of RSV, particularly in vulnerable populations, it is imperative to develop more accessible and effective therapeutic strategies beyond prophylactic measures.

Given as a prophylactic treatment specifically to infants and/or babies born prematurely, babies with heart disease, and children with bronchopulmonary dysplasia; palivizumab also known as Synagis is an example of the first approved mAb given to target RSV F protein [113]. Besides that, nirsevimab, also sold under the brand name Beyfortus is another mAb that was later approved by the U.S. FDA which helps prevent severe RSV in infants and young children [114]. However, both are mainly targeted at high-income countries, as the costs are expected to be prohibitively high for affordable global access to the product [115]. Moreover, despite the safety and moderate to good efficacy (approximately 50–70%) of palivizumab (Synagis) and nirsevimab—when used as prophylactic treatments, systemically administered mAbs have not been successful as a therapeutic option for RSV infections [113].

Another muco-trapping study of combating RSV was reported using an inhaled motavizumab variant, known as Mota-MT, which is a highly effective neutralizing mAb targeting the RSV F protein [72]. Findings from this study demonstrated Mota-MT’s efficacy in mice by capturing RSV within airway mucus through polyvalent Fc-mucin interactions, resulting in a 20–30-fold reduction in the movement of RSV particles in pediatric and adult mucus, dependent on the Fc-glycan mechanisms. Furthermore, Mota-MT rapidly eliminated the virus from the mouse airways. When administered daily via nebulization to RSV-infected neonatal lambs, the treatment achieved an astonishing reduction in RSV viral loads—10,000-fold in bronchoalveolar lavage fluid and 100,000-fold in lung tissues—compared to placebo controls [72]. The success of Mota-MT in preclinical models is particularly encouraging, suggesting that muco-trapping strategies could revolutionize the early intervention landscape for RSV infections. These substantial results demonstrated the immense potential of inhalable muco-trapping monoclonal antibodies as an innovative therapeutic strategy for RSV.

An antiviral study of nebulised ALX-0171, a novel trivalent nanobody with antiviral properties against RSV has been carried out which targets the F protein of RSV to block the virus from entering host cells [73–75]. When administered prophylactically or therapeutically through nebulization or intranasal delivery in cotton rats, ALX-0171 showed notable success in reducing viral loads. Its antiviral effect was more significant as compared to the systemic administration of palivizumab, an approved mAb for RSV treatment. A Phase I/IIa clinical trial found nebulized ALX-0171 was well-tolerated and effective in lowering nasal RSV viral titers among young children [74]. Taken together, these findings reflect a growing consensus that inhaled biologics represent a viable and potentially superior alternative to systemic prophylaxis for respiratory viral infections like RSV.

## Concluding remarks

Pulmonary drug delivery has evolved remarkably, shifting from traditional therapies to biologics-based approaches that target complex respiratory diseases. Inhalation therapies, once confined largely to small molecules for asthma and COPD, now show immense potential for delivering proteins, monoclonal antibodies, nanobodies, and even gene-based therapeutics. As the limitations of systemic biologic delivery become increasingly apparent, pulmonary administration offers a more localized, non-invasive and efficient therapeutic approach. It facilitates direct delivery to target site, enhances therapeutic efficacy and minimizes side effects, all while potentially improving patient adherence through ease of use.

These biologic agents exhibit distinct pharmacokinetic and pharmacodynamic profiles depending on their size and structure, necessitating tailored formulation and device strategies. Recent advances in formulation, such as PEGylation, encapsulation, and powder-based delivery, have helped to overcome challenges related to biologic stability and tissue penetration. Similarly, innovations in inhaler design from DPIs and pMDIs to smart inhalers and mesh nebulizers are making it increasingly feasible to deliver sensitive biologic molecules effectively and reproducibly to the lungs.

However, despite these encouraging advancements, challenges remain. Achieving consistent aerosol delivery, preserving biologic stability during nebulization, ensuring uniform lung distribution, and overcoming mucosal and immunological barriers still require significant optimization. Encouragingly, emerging technologies such as mucopenetrating formulations, breath-actuated devices, and digitally monitored inhalers have shown potential in addressing some of these issues. However, translating preclinical success into consistent clinical efficacy remains a key barrier.

Ultimately, interdisciplinary collaboration across immunology, pulmonary medicine, pharmaceutical sciences, and device engineering will be essential to unlock the full potential of inhaled biologics. As more targeted therapies enter development and regulatory pathways begin to adapt to these novel delivery systems, inhaled biologics may soon become a widely adopted strategy in the management of complex respiratory diseases.

## Data Availability

Data sharing not applicable to this article as no datasets were generated or analysed during the current study. All data mentioned were publicly available data that was cited in this paper.
