# Publisher Correction: Inhaled biologics for respiratory diseases: clinical potential and emerging technologies

**DOI:** 10.1007/s13346-025-01985-8

**Published:** 2025-09-27

**Authors:** Nur Adania Shaibie, Nur Dini Fatini Mohammad Faizal, Fhataheya Buang, Teerapol Srichana, Mohd Cairul Iqbal Mohd Amin

**Affiliations:** 1 Centre for Drug Delivery Technology and Vaccine, Faculty of Pharmacy, Universiti Kebangsaan Malaysia, Jalan Raja Muda Abdul Aziz, Kuala Lumpur, 50300 Malaysia; 2 Drug Delivery System Excellence Centre, Department of Pharmaceutical Technology, Faculty of Pharmaceutical Sciences, Prince of Songkla University, Hat Yai, 90112 Songkhla Thailand

**Drug Delivery and Translational Research**

10.1007/s13346-025-01909-6

The incorrect PDF of this article was originally published. It was corrected. The publisher regrets the error.

